# Bibliometric analysis of toll-like receptor agonists associated with cancer therapy

**DOI:** 10.1097/MD.0000000000028520

**Published:** 2022-01-07

**Authors:** Wei Li, Li Wan, Shaojun Duan, Jingjing Xu

**Affiliations:** a Department of Pharmacy, Maternal and Child Health Hospital of Hubei Province, Tongji Medical College, Huazhong University of Science and Technology, Wuhan, China; b Department of Pharmacy, Jincheng people's Hospital, Jincheng, Shanxi, China.

**Keywords:** agonist, bibliometric analysis, cancer, toll-like receptor, visualization

## Abstract

**Background:**

Toll-like receptors (TLRs), a family of innate pattern-recognition receptors, have been exploited as a target for antitumor strategy. An increasing number of TLR agonists, serving as immunotherapeutics or vaccine adjuvants, were developed. This study aimed at exploring the status and trend of current researches on TLR agonists through bibliometric analysis.

**Methods:**

Original publications on TLR agonists were collected from the Web of Science Core Collection. Data were analyzed in terms of publication outputs, journals, countries, institutions, authors, co-authorship, co-citation, research hotspots, and evolution trends through VOSviewer and CiteSpace.

**Results:**

A total of 1914 TLR agonists-related articles, published in 612 academic journals between 2000 and 2019, were enrolled in the study. The *Journal of Immunology* published the most publications, followed by *PLoS One* and *Blood*. The USA that is in possession of the largest number of articles and the most extensive cooperators was the most leading country in this field. University of Minnesota ranked the first in terms of paper totality, but its average citations ranking was lower than University of Pennsylvania. Gudkov AV was the most productive author, whose team reported a TLR5 agonist that had radioprotective activity in mouse and primate models in 2008. The paper of Akira Shizuo, professor of Osaka University, was widely cited by international peers. The research trend of TLR agonists has undergone 3 periods: mechanisms of TLR signalings in immunotherapy (2000–2010), discovery of TLR agonists (2011–2014), application, therapeutic evaluation, and drug design of TLR agonists (2015–2019).

**Conclusion:**

This study provides investigators a landscape of TLR agonists research from the perspective of bibliometrics.

## 1 Introduction

Toll-like receptors (TLRs), expressed by innate immune and tumor cells, are a family of innate pattern-recognition receptors including a series of receptors such as TLR1-TLR10. The activation of TLR can result in initiation of innate and adaptive immune responses, which can be exploited as a target for antitumor strategy. An increasing number of TLR agonists, serving as immunotherapeutics or vaccine adjuvants, are developed.^[1]^ For instance, Imiquimod, the first FDA-approved TLR7 agonist, is used for the treatment of superficial basal cell carcinoma.^[2]^ Two anticancer vaccines, Coley's toxin^[3]^ and Bacille Calmette-Guerin (BCG),^[4]^ are used individually as weak anticancer immunotherapy by targeting multiple TLRs. Although a series of progress has been made in the area of immunotherapy based on TLR agonists, the ineffectiveness or serious side effect of those agonists is still an urgent problem.^[1]^ To attenuate the dose-limiting toxicity of tested TLR agonists, clinical trials involving TLR agonists in combination with other antineoplastic agents have been approved.^[5,6]^ Except for antitumor effects, as a double-edged sword, TLR stimulation also plays pro-tumor effects.^[7]^ The driving factors of anti- and pro-tumor effects are still controversial. Most investigators believe that the pro-tumor effect is attributed to TLR expression by immune cells, while several studies indicate the pro-tumor effect is driven by TLR expression by tumor cells. In general, TLR agonists are promising anticancer drugs either as monotherapy or combinations with other ingredients.

Bibliometrics, different from systematic reviews, is often adopted to assess the tendency of research activity, quantitatively and qualitatively of literature in a certain time span based on published journal articles.^[8]^ Existing publications mainly summary TLR agonists in preclinical and clinical studies. However, there is no bibliometric analysis on this area. In this paper, we intend to use this statistical analysis to provide the landscape of TLR agonists in cancer therapy.

## 2 Methods

### 2.1 Data collection

Data were obtained from the Web of Science Core Collection (WoSCC) on November 11, 2019. The search query terms were as follows: TS= (“Toll-Like Receptor” OR TLR) AND TS= (agonist OR activator) AND TS= (cancer OR tumor OR neoplasm OR oncology OR sarcoma OR leukaemia OR leukemia OR lymphoma OR adenocarcinoma) AND Language: (English) AND Document Types: (Article). Time span was set to between 1900 and November 11, 2019.

### 2.2 Data extraction

The query retrieved 1914 records between 2000 and November 11, 2019. The full records and cited references option were selected, when data was downloaded from WoSCC. Tab-delimited file format was recommended for VOSviewer, while plain text file format was for CiteSpace. The bibliographic records, including title, abstract, authors, journals, institutions, countries, keywords, references, WoSCC categories, quartile in category and impact factor (IF) of the journal, were extracted.

### 2.3 Data analysis

VOSviewer 1.6.13 was used to analyze the journal citation, co-authorship, and co-citation network. CiteSpace 5.4 was performed to analyze the co-citation keywords. OriginPro 9.1 was applied to make histograms and line charts. All analyses were based on previous published studies, thus no ethical approval and patient consent are required.

## 3 Results

### 3.1 Publication outputs

**Figure 1:**
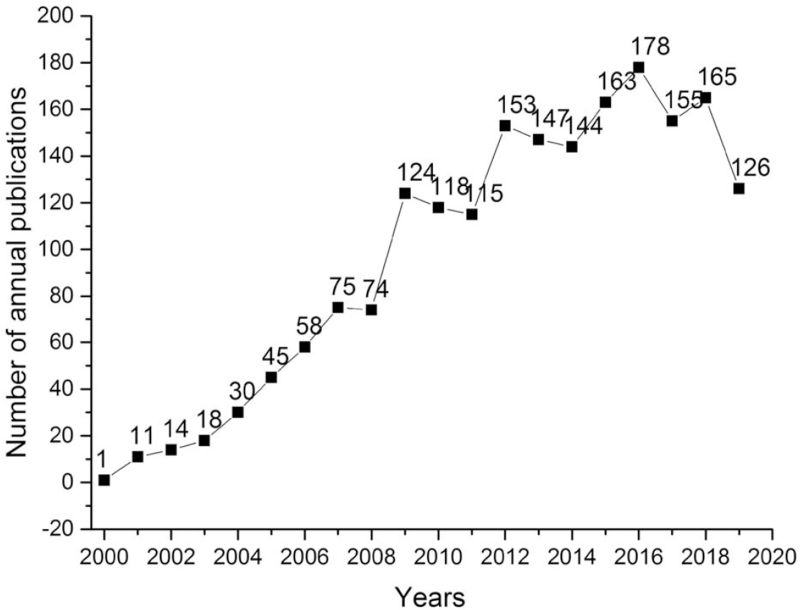
Annual publications on TLR agonists-related research. TLR = toll-like receptor.

A total of 1914 articles were included in this study, and the first article was published in 2000. The number of publications experienced rapid growth in 2008 and reached a peak in 2016. Except for a slight decline in 2019, which might be caused by the delayed publication, the overall trend of publication is volatile and rising (Fig. 1).

### 3.2 Journal analysis

The 1914 TLR agonists-related publications were published in 612 academic journals. Of the 612 journals, 80 journals met the threshold of a minimum of 5 publications, accounting for 13.07% of the total journal sources. And the top 10 active journals published 412 articles, accounting for 21.53% of the total (Table 1). With 73 counts, the *Journal of Immunology* (Impact Factor, IF2018, 4.539) published the most publications, accounting for 3.81%, followed by *PLoS One* (IF2018, 2.776) and *Blood* (IF2018, 16.562) (Table 1).

**Table 1:** Top 10 active journals.

Rank	Journal	Country	Counts	IF2018
1	*Journal of Immunology*	USA	73	4.539
2	*PLoS One*	USA	*55*	2.776
3	*Blood*	USA	43	16.562
4	*Infection and Immunity*	USA	*39*	3.160
5	*Cancer Research*	USA	*38*	8.378
6	*Cancer Immunology Immunotherapy*	USA	38	4.900
7	*Journal of Biological Chemistry*	USA	*38*	4.106
8	*Clinical Cancer Research*	USA	35	8.911
9	*Molecular Medicine Reports*	Greece	28	1.851
10	*Oncoimmunology*	USA	*25*	5.333

### 3.3 Country and institution analysis

The 1914 literatures were contributed by authors from 67 countries, mainly from the USA, China, Germany, Japan, UK, and France. The top 10 countries (5 European countries, 2 American countries, and 3 Asian countries) published most of the articles, which were pioneers in TLR agonists-related area. In terms of number of published articles, China with 338 literatures ranked the second. However, the ranking of average citations of China was inconsistent with that of the total published papers, which indicated that China should make efforts to improve the quality of papers.

2082 institutions contributed these 1914 TLR agonists-related studies. The top 10 institutions published 248 articles, accounting for 12.95% of the total. University of Minnesota (31 articles, USA) ranked the first, followed by University of California Los Angeles (27 articles, USA) and Harvard University (26 articles, USA) (Table 2). In the top 10 list, the USA institutions occupied 8 positions, which suggested its undisputed leadership in this research field. Even more striking, the University of Pennsylvania in possession of 23 publications topped the list in average citations.

**Table 2:** Ten leading countries and institutions.

Rank	Country	Frequency	Avg. citations	Institution	Frequency	Avg. citations
1	USA	789	42.3	Univ Minnesota	31	32.8
2	China	338	15.7	Univ Calif Los Angeles	27	47.9
3	Germany	170	46.7	Harvard Univ	26	43.1
4	Japan	151	40.2	Univ Maryland	26	39
5	UK	110	47.3	Radboud Univ Nijmegen	25	33.4
6	France	98	49.0	Univ Penn	23	101.5
7	South Korea	97	19.0	Univ Washington	23	46.6
8	Canada	77	29.7	Sungkyunkwan Univ	23	25.0
9	Netherlands	71	47.9	Univ Calif San Diego	22	59.3
10	Switzerland	66	48.1	China Med Univ	22	28.6

### 3.4 Author analysis

These 1914 literatures were drafted by 12,392 authors and the average number of co-authors per article was 6.47. Table 3 presented the list of top 20 productive authors. The top 20 prolific authors mainly came from the USA, China, and Italy. Gudkov AV (USA, 11 articles) topped the table, followed by Disis ML (USA, 10 articles), Balsari A (Italy, 10 articles), and Lu H (USA, 10 articles). Gudkov AV came from Roswell Park Comprehensive Cancer Center, whose team reported a TLR5 agonist that had radioprotective activity in mouse and primate models.^[9]^ He has extensive collaborators, such as Feinstein E (Israel), Haber M (Australia), and Naroditsky B (Russia). Burdelya LG, and the average citations of his colleague was 73.2, headed the table, followed by Gudkov AV (64.5), Yang Y (43.8), and Disis ML (35.7). Overall, the co-authorship connection was mainly restricted to national authors and the international cooperation remained to be strengthened.

**Table 3:** Top 20 prolific authors ranked by number of publications.

Author (country)	Frequency (citation)	Avg. citation	Author (country)	Frequency (citation)	Avg. citation
Gudkov AV (USA)	11 (710)	64.5	Liu SJ (China)	8 (100)	12.5
Disis ML (USA)	10 (357)	35.7	Chen W (China)	8 (198)	24.7
Balsari A (Italy)	10 (131)	13.1	Chuang JH (China)	8 (85)	10.6
Lu H (USA)	10 (321)	32.1	Salazar AM (USA)	8 (217)	27.1
Tagliabue E (Italy)	9 (113)	12.6	Yang Y (China)	7 (307)	43.8
Hershberg RM (USA)	9 (184)	20.4	Sfondrini L (Italy)	7 (89)	12.7
Burdelya LG (USA)	9 (659)	73.2	Sommariva M (Italy)	7 (92)	13.1
Bourquin C (Switzerland)	9 (193)	21.4	Dietsch GN (USA)	7 (168)	24
Endres S (Germany)	8 (219)	27.4	Cohen PA (USA)	7 (127)	18.1
Liu X (China)	8 (119)	14.9	Carson DA (USA)	7 (93)	13.3

### 3.5 Co-citation analysis

**Figure 2:**
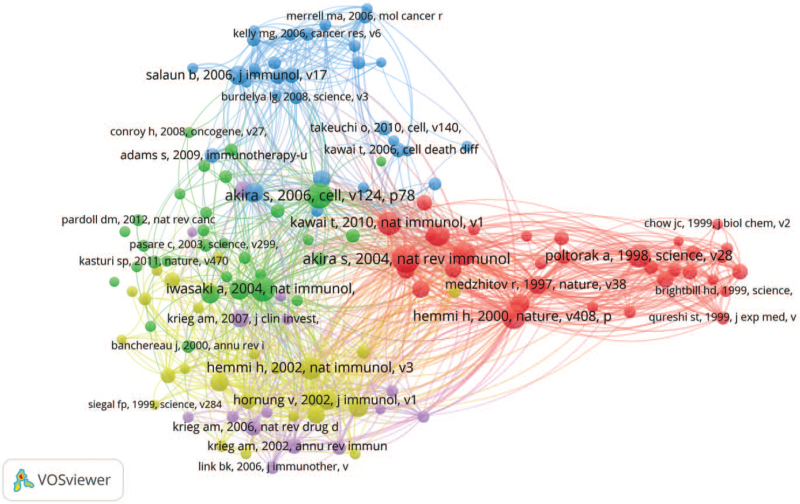
Cited reference co-citation network map.

Co-citation analysis was effective in establishing relationships among documents, which reflected the key concepts, methods, experiments, or evolution path in a field.^[10]^ The co-citation network consisted of 60,746 references with 137 nodes and 12,680 connection lines (Fig. 2). The red central node represented Akira Shizuo, professor of Osaka University, who had won several international awards for his outstanding contribution to the TLR related research. His article entitled “Toll-like receptor signaling” published in *Nat Rev Immunol,* systematically summarized the rapid progress in discovering molecular mechanisms that mediate TLR signaling.^[11]^ This article was cited 144 times all together in these 1914 TLR related literature and 1773 times in WoSCC, respectively. These top 10 cited references including 5 reviews and 5 articles, which were the intellectual bases in TLR research (Table 4 and Fig. 2). In particularly, 6 (4 reviews and 2 articles) of the top 10 cited literatures were published by Akira Shizuo's research group (Osaka University, Japan), manifesting Japan's unignorable influence in this field.

**Table 4:** Top 10 cited references in TLR research.

Rank	Title	Country	Year	Citations	Citations in WoSCC
1	Toll-like receptor signaling.	Japan	2004	144	5331
2	Pathogen recognition and innate immunity.	Japan	2006	119	6397
3	Toll-like receptor control of the adaptive immune responses.	USA	2004	108	2748
4	A Toll-like receptor recognizes bacterial DNA.	Japan	2000	104	4482
5	The role of pattern-recognition receptors in innate immunity: update on Toll-like receptors.	Japan	2010	99	4153
6	Recognition of double-stranded RNA and activation of NF-kappaB by Toll-like receptor 3.	USA	2001	96	3989
7	Small anti-viral compounds activate immune cells via the TLR7 MyD88-dependent signaling pathway.	Japan	2002	96	1630
8	Defective LPS signaling in C3H/HeJ and C57BL/10ScCr mice: mutations in Tlr4 gene.	USA	1998	96	5577
9	Toll-like receptors.	Japan	2003	95	3971
10	Selected toll-like receptor agonist combinations synergistically trigger a T helper type 1-polarizing program in dendritic cells.	Switzerland	2005	73	831

### 3.6 Keywords co-occurrence analysis and new research trends

High frequency keywords represented research hotspots during a period of time, whereas burst keywords uncovered new research trend.^[12]^ More than 7500 keywords were extracted from the titles and abstracts of the 1914 literatures. The top 20 high-frequency keywords were listed in Table 5. These keywords which covered the TLR signaling pathway investigation, immune response, immunotherapy, and therapeutic assessment were the research hotspots in this field. CiteSpace was employed to identify the global trend of research on TLR agonists by detecting burst keywords. The burst keywords that changed over time were characterized by the red line stream as shown in Table 6. The research trend had undergone 3 periods: mechanisms of TLR signalings in immunotherapy (2000–2010), discovery of TLR agonists (2011–2014), application, therapeutic evaluation, and drug design of TLR agonists (2015–2019).

**Table 5:** The top 20 co-occurrence keywords.

Rank	Keyword	Counts	Rank	Keyword	Counts
1	Toll like receptor	593	11	Tumor necrosis factor	169
2	Activation	396	12	In vivo	167
3	Dendritic cell	370	13	Cell	153
4	Expression	367	14	Response	152
5	NF-κB	277	15	Lipopolysaccharide	149
6	Inflammation	239	16	T cells	136
7	Cancer	215	17	Induction	127
8	Immunotherapy	193	18	Gene expression	124
9	Immune response	182	19	Apoptosis	114
10	Innate immunity	174	20	Recognition	111

**Table 6:**
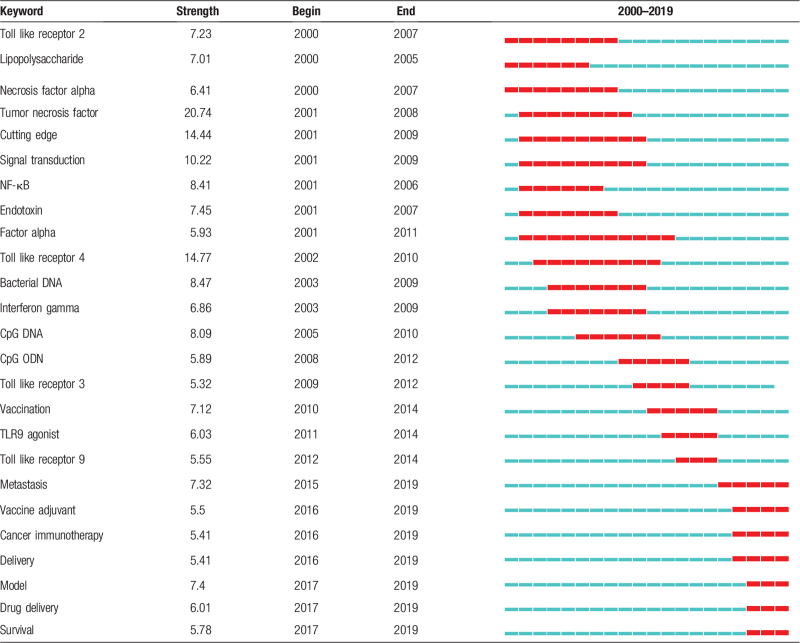
Top 25 keywords with the strongest citation bursts.

## 4 Discussion

Since TLR initially discovered as “Toll” in antifungal immunity of drosophila in 1996, numerous TLRs modulators such as synthetic agonists, microbial products, and endogenous ligands were undergone preclinical or clinical investigation, which resulted in a boom in paper output.^[13,14]^ The USA is the undisputed master in TLR related field, which can be attributed to its powerful innovation and technological strength, many well-known scientific research institutes, a great deal of research grants, and the world's largest gross domestic product. As the only developing country in the list of 10 leading countries in TLR, China is closely related to its uninterrupted funding for basic researches. However, the quality of its literature remains to be improved. In particular, Akira Shizuo's team (Osaka University, Japan) have done much basic research work in immunology, which has been widely cited by international peers, manifesting Japan's great influence in this field.

Even though the clinical achievements of TLR agonists are not as eye-catching as other immunotherapeutics including immune checkpoint blockers and chimeric antigens receptor-T cells, the combinatorial approaches provide an opportunity to boost the immune response resulting in better clinical outcomes. Several combinatorial approaches such as TLR agonists with radiotherapy, chemotherapy, and monoclonal antibodies have been tested.^[15–18]^ Though some TLR agonists exhibit antitumor efficacy, the pro-tumor efficacy of those should not be ignored.^[7]^ The exact antitumor mechanisms of those agonists remain to be further investigated.

There were some limitations in this study. First, a small number of publications were not included in this study since the searched database was limited to Web of Science. Second, for China, due to language differences, many scientists’ papers cannot be published in English, which cannot reflect the true level of China in this field.

In conclusion, a total of 1914 TLR agonists-related articles, published in 612 academic journals, were included in this study. Overall, the number of publications has been going up year by year from 2000 to 2019. The *Journal of Immunology* published the most publications, followed by *PLoS One* and *Blood*. The USA, which was the most leading country in this field, in possession of the largest number of articles and the most extensive cooperators. The University of Minnesota ranked the first in terms of the total number of papers, but its average citations ranking was lower than the University of Pennsylvania. Gudkov AV, was the most productive authors, whose team reported a TLR5 agonist that had radioprotective activity in mouse and primate models in 2008. Akira Shizuo is a professor of Osaka University, whose paper had been widely cited by international peers. The research trend of TLR agonists has undergone 3 periods: mechanisms of TLR signalings in immunotherapy (2000–2010), discovery of TLR agonists (2011–2014), application, therapeutic evaluation, and drug design of TLR agonists (2015–2019). In summary, this study gives investigators the landscape of TLR agonists research from the perspective of bibliometrics.

## Author contributions

**Data curation:** Li Wan, Shaojun Duan, Jingjing Xu.

**Formal analysis:** Li Wan, Shaojun Duan, Jingjing Xu.

**Methodology:** Wei Li, Jingjing Xu.

**Software:** Wei Li, Jingjing Xu.

**Validation:** Wei Li, Li Wan, Shaojun Duan, Jingjing Xu.

**Writing – original draft:** Li Wan, Shaojun Duan, Jingjing Xu.

**Writing – review & editing:** Wei Li, Jingjing Xu.
